# Peptide vaccine for semaphorin3E ameliorates systemic glucose intolerance in mice with dietary obesity

**DOI:** 10.1038/s41598-019-40325-y

**Published:** 2019-03-07

**Authors:** Yohko Yoshida, Ippei Shimizu, Yuka Hayashi, Ryutaro Ikegami, Masayoshi Suda, Goro Katsuumi, Takayuki Wakasugi, Masaaki Nakao, Hironori Nakagami, Ryuichi Morishita, Tohru Minamino

**Affiliations:** 1 Department of Cardiovascular Biology and Medicine, Niigata University Graduate School of Medical and Dental Sciences, Niigata, Japan; 2 Division of Molecular Aging and Cell Biology, Niigata University Graduate School of Medical and Dental Sciences, Niigata, Japan; 3 Department of Health Development and Medicine, Osaka University Graduate School of Medicine, Osaka, Japan; 4 Department of Clinical Gene Therapy, Osaka University Graduate School of Medicine, Osaka, Japan

## Abstract

We previously demonstrated that cellular aging signals upregulated a secreted class 3 semaphorin E (Sema3E) and its receptor plexinD1 in the adipose tissue of a murine model of dietary obesity and that Sema3E was a chemoattractant, mediating its biological effects by inducing infiltration of plexinD1-positive inflammatory macrophages into the visceral white adipose tissue. This study was performed to develop a peptide vaccine for Sema3E and test its therapeutic potential in a murine model of dietary obesity. Two antigenic peptides were selected to generate neutralizing antibodies for a vaccine. These peptides were conjugated to keyhole limpet hemocyanin (KLH), and were administered with Freund’s adjuvant to obese wild-type male mice. The Sema3E antibody titer was analyzed by ELISA, and the biological effects of the peptides were tested in mice with dietary obesity. Among the two candidate peptides, the Sema3E antibody titer was significantly increased by injection of KLH-conjugated HKEGPEYHWS (Sema3E vaccine). Administration of Sema3E vaccine suppressed the infiltration of plexinD1-positive cells, ameliorated chronic inflammation in visceral white adipose tissue, and improved systemic glucose intolerance in mice with dietary obesity, suggesting that Sema3E vaccine has the potential to become a next generation therapy for obesity and diabetes.

## Introduction

The number of patients with diabetes continues to increase, so it is an urgent task to find better therapies for this disorder. Chronic sterile inflammation of visceral white adipose tissue (WAT) develops under metabolic stress, and is well accepted to have a causal role in the development and progression of systemic metabolic disorders^1,2^. We previously demonstrated that cellular aging signals were activated in visceral WAT by metabolic stress and contributed to adipose tissue inflammation^3^. We further found that cellular aging signals upregulated a secreted class 3 semaphorin E (Sema3E) and its receptor plexinD1 in visceral WAT of a murine model of dietary obesity^4^. Semaphorins and their receptors (plexins) are axon-guiding molecules that regulate development of the nervous system during embryogenesis. We found that Sema3E is a chemoattractant, which mediates its biological effects by promoting the infiltration of plexinD1-positive inflammatory macrophages into visceral WAT under metabolic stress. In addition to mice with dietary obesity, the circulating Sema3E level is also increased in patients with diabetes, suggesting that suppression of this secreted molecule could become a next generation therapy for diabetes by inhibiting chronic inflammation in visceral WAT^4^. Inhibiting Sema3E by administration of a neutralizing antibody is one option, but the diabetic population is huge and the expected medical cost burden is very large, so we considered other strategies for targeting this molecule. Accordingly, we tried to generate a peptide vaccine in order to utilize the endogenous immune system for production of Sema3E neutralizing antibody. In the present study, we demonstrated that a peptide vaccine targeting Sema3E could suppress inflammation in visceral WAT and improve glucose intolerance in mice with dietary obesity.

## Results

### Sema3E-vaccine inhibits adipose tissue inflammation in visceral fat

**Figure 1:**
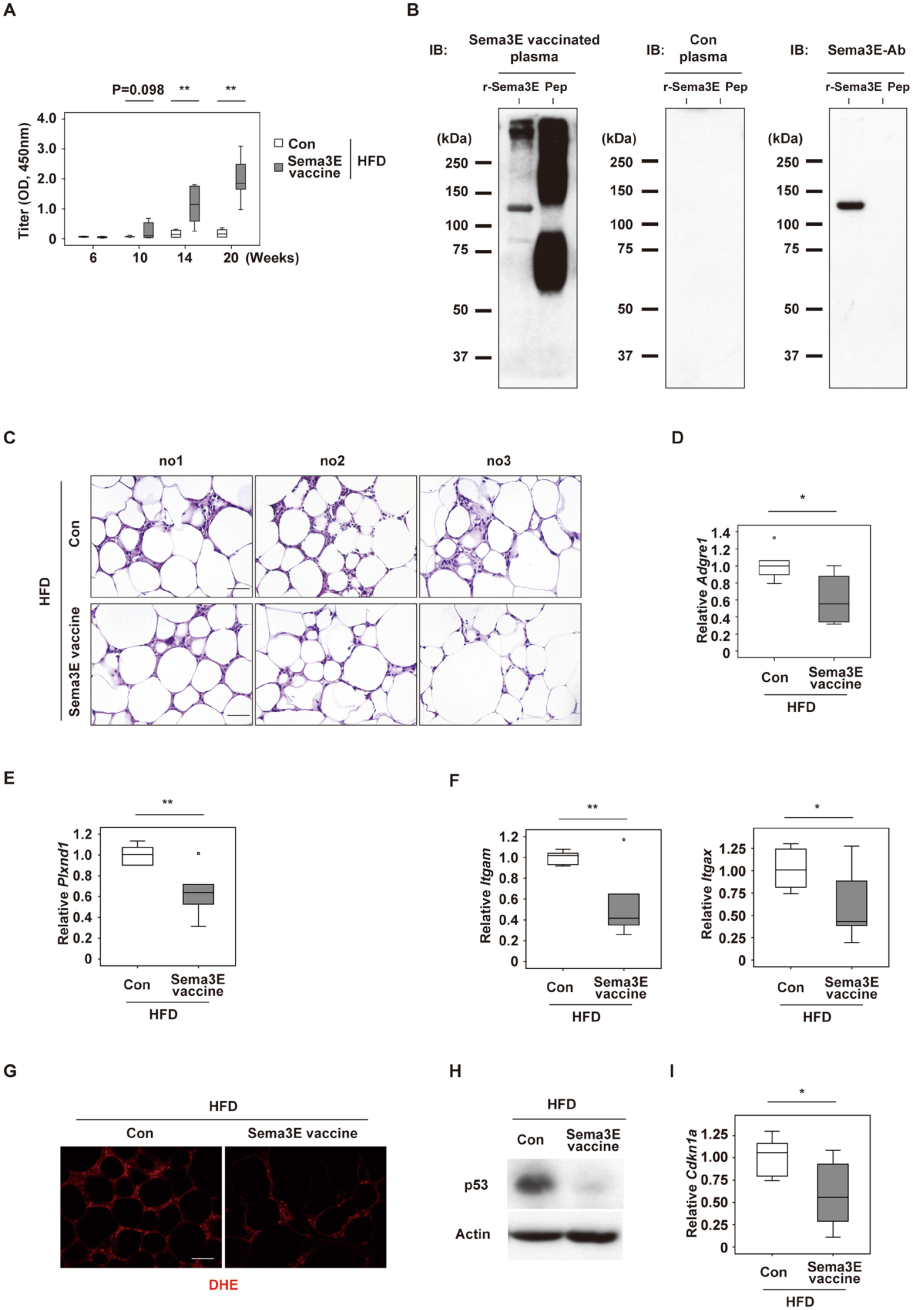
Sema3E vaccine inhibits inflammation in visceral fat. (**A**) Mice fed a high fat diet (HFD) were immunized with KLH-conjugated HKEGPEYHWS peptide (Sema3E vaccine group) or KLH alone (KLH group), and plasma antibody titers were analyzed with ELISA at 6, 10, 14 and 20 weeks of age (n = 4, 4, 4, 4, 4, 4, 4, 4). (**B**) After the third dose, plasma was collected from the Sema3E vaccine group and the KLH group (Con). Recombinant Sema3E (r-Sema3E) protein or BSA-conjugated Sema3E peptide (Pep) was blotted with plasma samples or with a commercially available anti-Sema3E antibody (Sema3E-Ab). (**C**) Hematoxylin and eosin (HE) staining of epididymal WAT (eWAT) harvested from mice in the Sema3E vaccine group (Sema3E vaccine) or the KLH group (Con) maintained on a HFD. Nos. 1, 2, and 3 indicate that the eWAT originated from different mice. Scale bar = 50 μm. (**D**–**F**) Levels of transcripts for *Adgre1* (Emr1)(D) (n = 6, 6), *Plxnd1* (E) (n = 6, 6), *Itgam* (n = 6, 6), and *Itgax* (n = 6, 6)(F) in the indicated groups. (**G**) Evaluation of reactive oxygen species (ROS) performed by dihydroethidium (DHE) staining in the indicated groups. Scale bar = 50 μm. (**H**) Western blot analysis of p53 in the indicated groups. Full-length blots are presented in Supplementary Fig. 2F. (**I**) *Cdkn1a* expression was examined in the indicated groups (n = 6, 6). Outliers were excluded by boxplot analysis. All remaining data were analyzed by the two-tailed Student’s *t*-test. *P < 0.05, **P < 0.01. Values represent the mean ± s.e.m. NS = not significant.

We tried to develop immunotherapy targeting Sema3E by injection of a Sema3E peptide vaccine to increase the level of circulating antibodies against Sema3E. Based on previous reports^5^, two antigenic peptides were selected to generate neutralizing antibodies targeting amino acids 385–394 (KVNGGKYGTT) or amino acids 359–368 (HKEGPEYHWS) of Sema3E. The N-terminus or lysine of each candidate peptide was conjugated to KLH via glutaraldehyde, and the synthetic peptides were purified by reverse-phase high-performance liquid chromatography (>99% purity)^6^. Then the KLH-conjugated peptides combined with Freund’s adjuvant were administered to wild-type male mice on a C57BL6/NCr background and the Sema3E antibody titer was analyzed by ELISA. In the chow fed mice, injection of the KLH-conjugated HKEGPEYHWS peptide led to a significant increase of the Sema3E antibody titer, while there was no marked increase after injection of the KLH-conjugated KVNGGKYGTT peptide (Supplementary Fig. 1A,B). Therefore, we further characterized the biological effects of the KLH-conjugated HKEGPEYHWS peptide (Sema3E vaccine) in mice with dietary obesity. Wild-type male mice were fed a high fat diet (HFD) from 4 weeks old and were administered 3 doses of Sema3E vaccine (at 6, 10, and 14 weeks old), followed by analysis at 20 weeks old (Supplementary Fig. 1C). A titer for sema3E increased mildly at 10 weeks of age after the initial injection. This increase became more significant after the second injection (at 14 weeks of age), and a robust increase was found at 20 weeks of age after the third injection (Fig. 1A). Antibodies in plasma from the Sema3E vaccine group bound to recombinant Sema3E protein and BSA-conjugated Sema3E peptide, unlike plasma from control mice (injected with KLH and Freund’s adjuvant) (Fig. 1B, left and middle panels). A commercially available anti-sema3E antibody detected recombinant Sema3E protein, but did not bind with BSA-conjugated Sema3E peptide (Fig. 1B, right panel). Infiltration of mononuclear-like cells and formation of crown-like structures in epididymal visceral WAT (eWAT) were note in mice with dietary obesity, while such changes were ameliorated by administration of Sema3E vaccine (Fig. 1C). There was a significant reduction in the expression of transcripts for *Adgre1* (Emr1), a macrophage marker (Fig. 1D). We previously reported that Sema3E is a chemoattractant, which mediates inflammatory effects by promoting infiltration of plexinD1-positive inflammatory macrophages into eWAT. We also found that expression of transcripts for plexinD1 was reduced by Sema3E vaccine therapy, as shown by quantitative PCR (qPCR) (Fig. 1E). We also found that the number of plexinD1-positive cells was reduced in visceral adipose tissue of the Sema3E vaccine group compared with the control group (Supplementary Fig. 2A). Furthermore, expression of markers for inflammatory macrophages, including *Itgam* and *Itgax* (CD11c), was reduced by the vaccine (Fig. 1F). Under this condition, qPCR studies showed transcripts for other immune cell markers including *Cd4*, *Cd8*, *Cd19*, *Cd335* and *Ly6g*, and a chemoattractant *Ccl2* were comparable between the two groups (Supplementary Fig. 2B,C). It is now well recognized that cellular senescence associated with elevated production of reactive oxygen species (ROS) promotes inflammation in inflamed visceral WAT^3,4,7,8^. We found that injection of Sema3E vaccine suppressed ROS production (Fig. 1G, and Supplementary Fig. 2D), and also reduced expression of p53 (a key mediator of cellular senescence) and its downstream molecule *Cdkn1a* (p21) (Fig. 1H,I and Supplementary Fig. 2E,F) in eWAT under metabolic stress. These results suggested that suppressing the infiltration of plexinD1-positive cells by administration of Sema3E vaccine contributed to inhibition of the vicious cycle between ROS production/cellular senescence and inflammation.

### Sema3E-vaccine ameliorates systemic glucose intolerance

**Figure 2:**
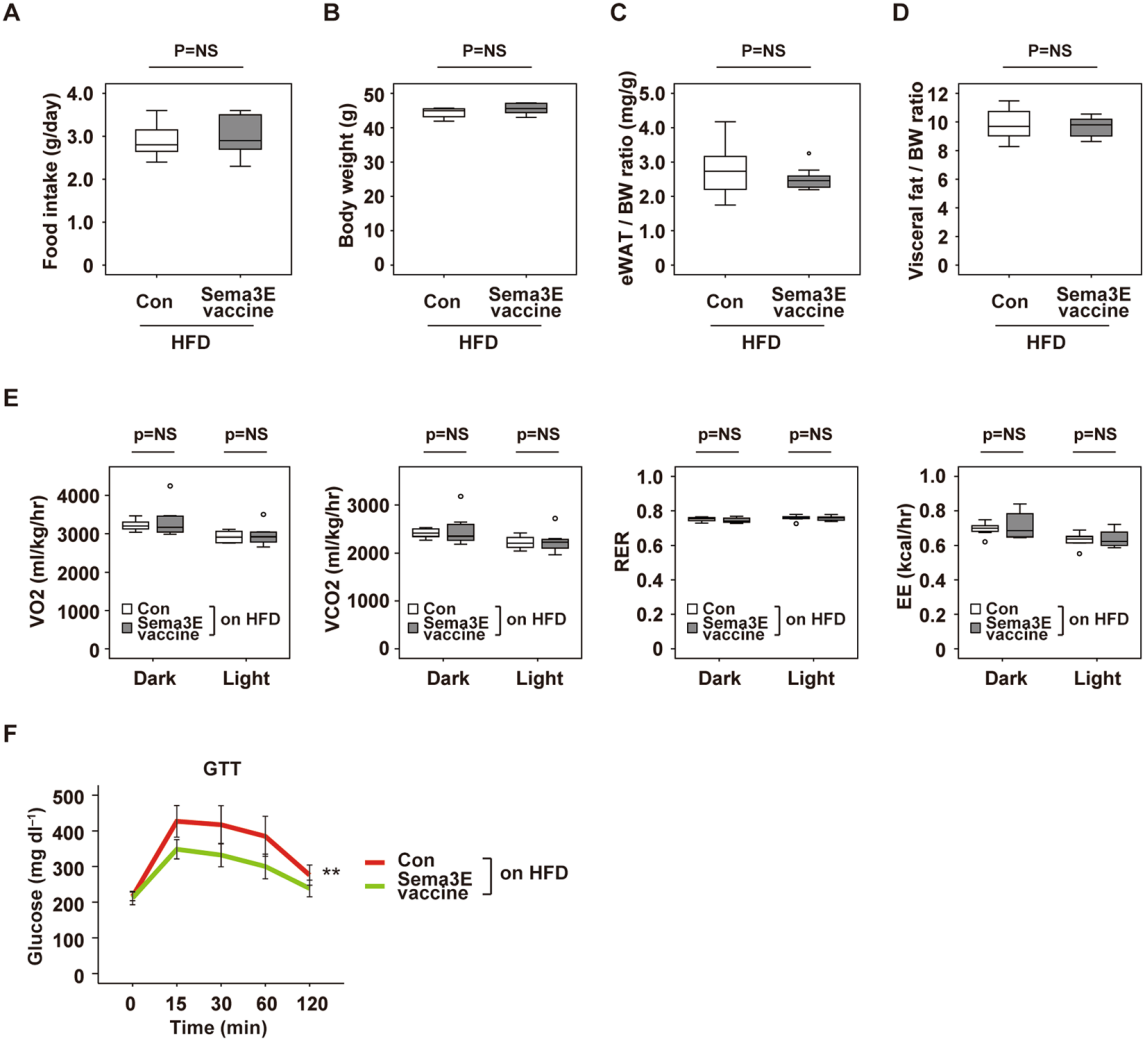
Sema3E vaccine improves systemic glucose intolerance. (**A**–**C**) Food intake (**A**) (n = 7, 6), body weight (**B**) (n = 6, 6), and eWAT/BW ratio (mg/g) (**C**) (n = 14, 11) of mice administered KLH-conjugated Sema3E vaccine (Sema3E vaccine) or KLH alone (Con). (**D**) Visceral fat/BW ratio of the indicated groups determined by CT scanning (n = 8, 8). (**E**) Oxygen consumption (VO_2_) (n = 8, 8), CO_2_ production (VCO_2_) (n = 8, 8), respiratory exchange ratio (RER) (n = 8, 8), and energy expenditure (EE) (n = 8, 8) in the indicated groups. (**F**) Glucose tolerance test (GTT) in the indicated groups (n = 14, 15). Outliers were excluded by boxplot analysis. All remaining data were analyzed by the two-tailed Student’s *t*-test (**A**–**E**) or repeated measures (**F**). *P < 0.05, **P < 0.01. Values represent the mean ± s.e.m. NS = not significant.

It is generally accepted that chronic sterile inflammation of visceral adipose tissue has a causal role in inducing systemic metabolic dysfunction^1,2^. Therefore, we tested the effects of Sema3E vaccine on systemic metabolism in mice with dietary obesity. We found that there were no differences between the control group and the Sema3E vaccine group with regard to food intake (Fig. 2A), body weight (Fig. 2B), eWAT weight (Fig. 2C), and visceral fat volume evaluated by CT (Fig. 2D). Metabolic cage studies showed that oxygen consumption (VO_2_), CO_2_ production (VCO_2_), the respiratory exchange ratio (RER), and energy expenditure (EE) were comparable between the control group and the Sema3E vaccine group (Fig. 2E). However, systemic glucose tolerance was better in the Sema3E vaccine group according to the glucose tolerance test (Fig. 2F), indicating that the vaccine improved systemic metabolic health as well as suppressing inflammation in visceral WAT.

### Characterization of the immune response to Sema3E-vaccine

**Figure 3:**
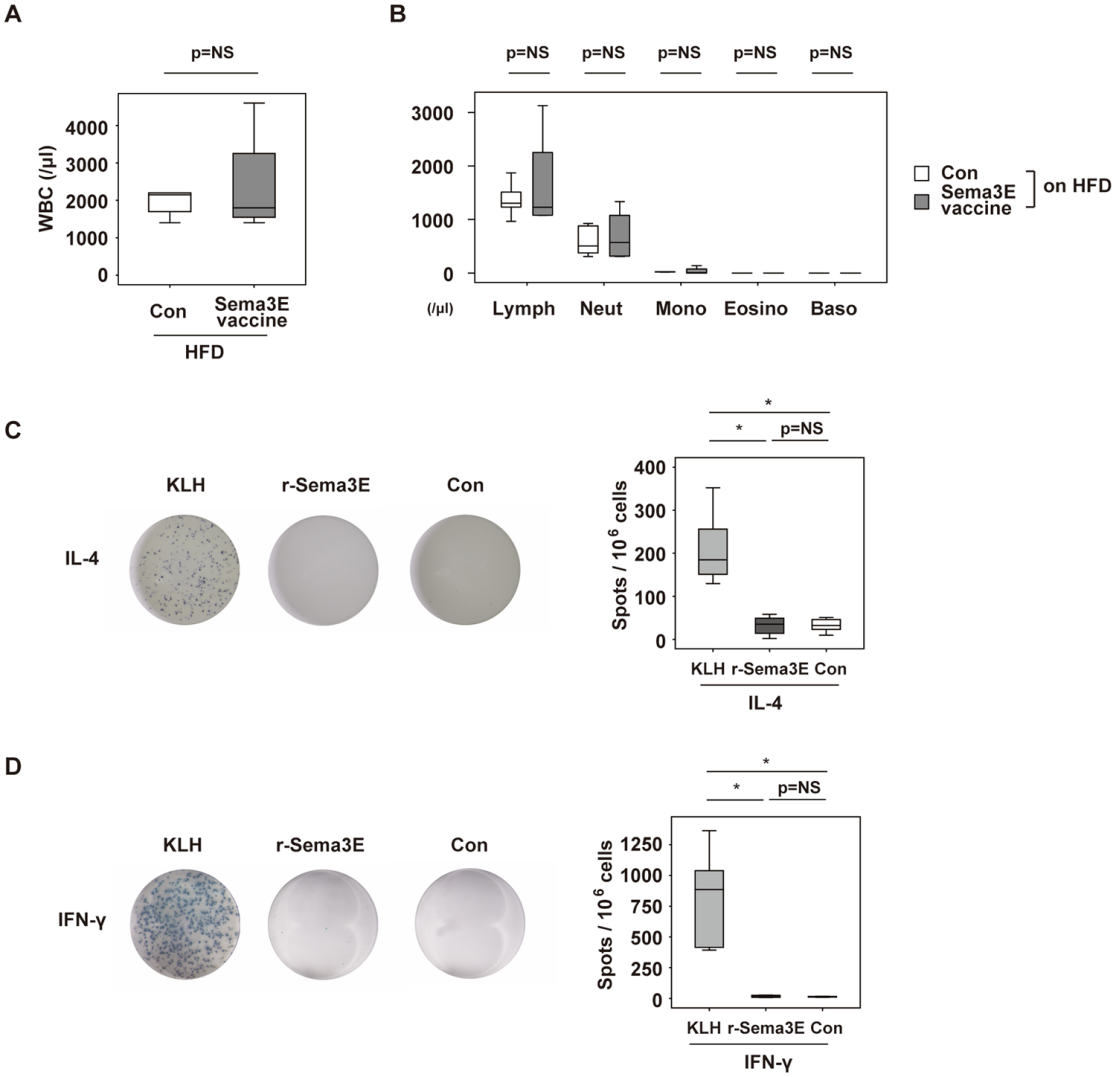
Characterization of immune response to Sema3E vaccine. (**A**) Peripheral white blood cell count (WBC) in mice administered KLH-conjugated Sema3E vaccine (Sema3E vaccine) or KLH alone (Con) (n = 6, 5) and maintained on a high fat diet (HFD). (**B**) Peripheral blood counts of lymphocytes (Lymph), neutrophils (Neut), monocytes (Mono), eosinophils (Eosino), and basophils (Baso) in the indicated groups (n = 6, 5). (**C**,**D**) ELISPOT assay showing production of IL-4 (**C**) or IFN-γ (**D**) by splenocytes harvested from mice fed an HFD and administered KLH-conjugated Sema3E vaccine (Sema3E vaccine). Splenocytes (10^6^ cells) were stimulated with KLH, recombinant Sema3E protein (r-Sema3E), or PBS (Con). Right panels indicate the number of spots per 10^6^ cells in the indicated groups (n = 6, 6, 6 for (**C**), n = 6, 6, 6 for (**D**)). No outliers or abnormal values were excluded by boxplot analysis. All data were analyzed by the two-tailed Student’s *t*-test (A, B) or 2-way ANOVA followed by Tukey’s multiple comparison test (**C**,**D**). *P < 0.05, **P < 0.01. Values represent the mean ± s.e.m. NS = not significant.

To investigate the potential immune response to Sema3E vaccine, we analyzed circulating immune cells, but found no differences between the groups (Fig. 3A,B). The enzyme-linked immunospot (ELISpot) test was performed using splenocytes from the Sema3E vaccine group to characterize T cell reactivity. Treatment with KLH led to an increase of IL-4 or IFN-γ production by the splenocytes, while recombinant Sema3E protein had no effect in this test (Fig. 3C,D). These results indicated that KLH contains epitopes for T cells, as reported previously^5^, while Sema3E protein did not elicit an immune response from T cells harvested from vaccinated mice. Taken together, these results suggest that a peptide vaccine targeting Sema3E could potentially become a therapeutic option for diabetes and/or unhealthy obesity in the future.

## Discussion

There is accumulating evidence about the role of cellular senescence in promoting the pathology of various age-related disorders, including diabetes, heart failure, and atherosclerosis^3,4,7–10^. We previously reported that accumulation of ROS, DNA damage, and telomere dysfunction under metabolic stress promotes p53-induced cellular senescence in visceral WAT, resulting in chronic sterile inflammation of this tissue that leads to development of systemic metabolic dysfunction^3^. Thus, elimination of senescent cells is a therapeutic option to be considered, and modulation of various molecules involved in cellular senescence is also an important field for exploration^9,11–13^. It can be predicted that direct targeting of p53 will lead to tumorigenesis^14^, so we have instead tried to suppress the p53-induced inflammatory response by targeting downstream molecules. We previously demonstrated that a secreted semaphorin, Sema3E, was positively regulated by p53 at the transcriptional level and was a chemoattractant for inflammatory macrophages expressing its receptor (Plexin D1). Suppression of the Sema3E-Plexin D1 axis contributed to inhibiting the inflammatory response in visceral WAT, resulting in improvement of systemic metabolic health. In addition, the circulating level of Sema3E was increased in patients with diabetes, indicating that this molecule could be a target of next generation therapy for diabetes^4^. Generation of an antibody directed against secreted Sema3E might be a promising approach, but considering the huge population of patients with unhealthy obesity and/or diabetes, we considered it crucial to develop a therapy that was also reasonable in terms of medical economy. Accordingly, we investigated the development of a peptide vaccine for Sema3E. Peptide vaccine therapy has already been shown to be safe and useful in the fields of hypertension and diabetes, targeting angiotensin II (AngII) and dipeptidyl peptidase-4 (DPP4), respectively. Vaccination utilizes the endogenous immune system to generate neutralizing antibodies for target molecules. Antigen-presenting cells (APCs) phagocytose the antigen-KLH conjugate and present an epitope of KLH to T cells via the major histocompatibility complex (MHC), leading to T cell activation. Then B cells that specifically recognize the target antigen differentiate into plasmacytes and proliferate with the support of activated T cells, followed by initiation of antibody production against antigens such as AngII or DPP4^6,15^. Importantly, this response can be achieved without induction of cell-mediated autoimmunity^5^. Using the same system, and this time Sema3E as an antigen, we tried to inhibit the Sema3E-PlexinD1 axis. We found that administration of a peptide vaccine based on amino acids 359–368 (HKEGPEYHWS) of Sema3E led to an increase of circulating antibodies and suppression of mononuclear-like cell infiltration into eWAT along with reduced Plexin D1 signaling. Our previous study revealed that Plexin D1 was expressed by inflammatory macrophages and was involved in systemic glucose intolerance. In the present study, we could show that administration of Sema3E vaccine suppressed the infiltration of these cells into eWAT and led to improvement of systemic metabolic health. Accordingly, inhibition of the Sema3E-Plexin D1 axis by vaccination may potentially be developed as a treatment for unhealthy obesity and diabetes.

## Materials and Methods

### Design and synthesis of the vaccine

To generate neutralizing antibodies for Sema3E, two antigenic peptide sequences (spanning amino acids 385–394 and amino acids 359–368 of Sema3E) were selected on the basis of previous reports (Pang Z, *et al*.^5^). The N-terminus or lysine of each candidate peptide was conjugated to keyhole limpet hemocyanin (KLH) via glutaraldehyde, and the synthetic peptides were purified by reverse-phase high-performance liquid chromatography (>99% purity) (Peptide Institute Inc., Osaka, Japan), as described previously^6^.

### Animal models

All animal experiments were conducted in compliance with the protocol reviewed by the Institutional Animal Care and Use Committee of Niigata University and approved by the President of Niigata University. C57BL/6NCr mice were purchased from SLC Japan (Shizuoka, Japan), and were fed a high fat diet (HFD) (CLEA Japan, Tokyo, Japan) or normal chow from 4 to 20 weeks of age. Each KLH-conjugated antigenic peptide (20 µg) and an equal volume of complete or incomplete Freund’s adjuvant (Sigma-Aldrich: F5881 (complete) or F5506 (incomplete)) were emulsified by vortexing. For immunization, mice were injected subcutaneously with the KLH-conjugated peptide at the age of 6 weeks (with complete Freund’s adjuvant), 10 weeks (with incomplete Freund’s adjuvant), and 14 weeks (with incomplete Freund’s adjuvant). Control groups received an equal volume of KLH mixed with complete or incomplete Freund’s adjuvant. Plasma was collected from the tail vein of each mouse for analysis of antibody titers.

### Antibody titer measurement

The antibody titer generated by injection of each peptide was measured by ELISA. A 96-well microtiter plate (Nunc-Immuno MicroWell 96 well solid plate, Thermo Scientific) was coated with one of the antigenic peptides conjugated to bovine serum albumin (BSA) in carbonate buffer. After coating, plasma collected from immunized mice was added to the wells and incubated overnight. Then the plate was incubated with the secondary antibody (ECL Anti-mouse IgG, Horseradish Peroxidase linked whole sheep antibody, GE Healthcare). After reaction with the TMB substrate (Sigma-Aldrich, T0440), the optical density was measured at 450 nm (OD450) by using a microtiter plate reader (iMark microplate reader, Bio-Rad).

### Physiological analyses

Mice were housed individually for monitoring of their body weight and food intake. Adiposity was examined by CT scanning (LaTheta, Aloca) according to the manufacturer’s protocol. CT scans were obtained at 2-mm intervals from the diaphragm to the base of the abdominal cavity. Oxygen consumption was measured in 20-week-old mice by using an O_2_/CO_2_ metabolic measurement system (Columbus Instruments) according to the manufacturer’s instruction.

### Laboratory tests

The intraperitoneal glucose tolerance test (IGTT) was performed as described previously with slight modifications, and glucose was given intraperitoneally at a dose of 1 g kg^−1^ (body weight)^4^. Peripheral white blood cells from immunized mice were counted by Oriental Yeast Co.(Tokyo, Japan).

### Histological analyses and immunostaining

Samples of epididymal white adipose tissue (WAT) were harvested, fixed overnight in 10% formalin, embedded in paraffin, and sectioned for immunofluorescence or hematoxylin-eosin (HE) staining. Staining for reactive oxygen species (ROS) was done, as described previously^4,7^. For immunostaining, deparaffinized sections were retrieved with citrate buffer and incubated with anti-PlexinD1 antibody (Abcam, ab28762) at 1:50 dilution. Anti-Goat IgG Cy5-conjugated (Abcam, ab6566) was used as a secondary antibody. The sections were stained with Wheat Germ Agglutinin, Alexa Fluor 488 Conjugate (Invitrogen, W11261, 1:10) and Hoechst (Life Technologies, 33258, 1:1000), and photographed with a Biorevo (Keyence Co., Osaka, Japan).

### RNA analysis

Extraction of RNA and real-time PCR (qPCR) was performed, as described previously^4^, using the following primers. *Rplp0* was used as the internal control.

*Adgre1*: GGAGGACTTCTCCAAGCCTATT, AGGCCTCTCAGACTTCTGCTT

*Ccl2:* CATCCACGTGTTGGCTCA, GATCATCTTGCTGGTGAATGAGT

*Cd4:* ACACACCTGTGCAAGAAGCA, GCTCTTGTTGGTTGGGAATC

*Cd8:* CTCACCTGTGCACCCTACC, ATCCGGTCCCCTTCACTG

*Cd19:* AAGGTCATTGCAAGGTCAGC, CTGGGACTATCCATCCACCA

*Cd335:* ACACTACTCATCACAGGAGGTGTT, GTTGAAAGGTCAAACTCCCAAT

*Cdkn1a*: TCCACAGCGATATCCAGACA, GGACATCACCAGGATTGGAC

*Itgam*: CAATAGCCAGCCTCAGTGC, GAGCCCAGGGGAGAAGTG

*Itgax*: ATGGAGCCTCAAGACAGGAC, GGATCTGGGATGCTGAAATC

*Ly6g:* GGCTCAGAAAAGTGCACCA, CGTACGTGGAAGCGAACAG

*Plxnd1*: CTGGATGTCCATCTGCATGT, CAGGAAGAACGGCTCACCTA

*Rplp0*: GATGCCCAGGGAAGACAG, ACAATGAAGCATTTTGGATAA.

### Western blot analysis

Western blot analysis was performed as described previously^4^. The primary antibodies were anti-p53 (1C12) antibody (Cell Signaling, #2524) and anti-actin antibody (Cell Signaling, #4970), which were used at a dilution of 1:1000. For western blot analysis of mouse plasma, recombinant full-length mouse Sema3E protein (R&D, 3238-S3) and the antigenic peptide conjugated with BSA were resolved in the buffer (10 mM Tris-HCl, pH 7.8, 150 mM NaCl, 1 mM EDTA) and subjected to SDS-PAGE. The membrane was incubated with plasma collected from mice immunized with the antigenic peptide or KLH (1:200 dilution) or with anti-Sema3E antibody (Santa Cruz, sc-49733) (1:1000 dilution) as the primary antibody, followed by the incubation with horseradish peroxidase-conjugated anti-mouse immunoglobulin G (Jackson Immunoresearch, #115-035-003) or anti-goat immunoglobulin G (Jackson Immunoresearch, #705-035-003).

### ELISPOT assay

ELISPOT assays for INF-γ and IL-4 were performed using Mouse IFN-γ ELISpot^PLUS^ and Mouse IL-4 ELISpot^PLUS^ (MABTECH Inc., 3321-4HST-2 for IFN-γand 3311-4HPW-2 for IL-4) according to the manufacturer’s instruction. Briefly, splenocyte suspensions from immunized mice were added to the 96-well ELISpot assay plates (10^6^ cells per well) and stimulated with 10 μg/mL recombinant mouse Sema3E protein (R&D, 3238-S3), 10 μg/mL KLH (WAKO, 080-07666), or PBS (Con) at 37 °C for 48 hrs. The plates were washed with PBS and incubated with biotin-conjugated detection antibodies for 2 hrs at room temperature. After washing, the plates were incubated with HRP-conjugated streptoavidin for 1 hr at room temperature, followed by the incubation with TMB substrate. Colored spots were photographed using a dissecting microscope and counted using ImageJ software.

### Statistical analysis

Statistical analysis was done with SPSS software (version 20). Results are shown as the mean ± SEM. Outliers were excluded by boxplot analysis. Differences between groups were examined by the two-tailed Student’s *t*-test, by repeated measures for temporal studies (glucose tolerance test), or 2-way ANOVA followed by Tukey’s multiple comparison test. In all analyses, *P* < 0.05 was considered statistically significant.

## Supplementary information

Supplementary Information
