# Supplementary material for: Peptide vaccine for semaphorin3E ameliorates systemic glucose intolerance in mice with dietary obesity
Source: Sci Rep. 2019 Mar 7;9:3858. doi: 10.1038/s41598-019-40325-y (PMC6405888; doi:10.1038/s41598-019-40325-y)
Supplement: Supplementary file 1 — Supplementary Information [file 41598_2019_40325_MOESM1_ESM.pdf]

Supplementary Information

Peptide vaccine for semaphorin3E ameliorates systemic glucose intolerance in mice with dietary obesity

Yohko Yoshida[1,2]*, Ippei Shimizu[1,2]*, Yuka Hayashi[1], Ryutaro Ikegami[1], Masayoshi Suda[1], Goro Katsuumi[1], Takayuki Wakasugi[1], Masaaki Nakao[1], Hironori Nakagami[3], Ryuichi Morishita[4], Tohru Minamino[1]**

[1]Department of Cardiovascular Biology and Medicine, Niigata University Graduate School of Medical and Dental Sciences

[2]Division of Molecular Aging and Cell Biology, Niigata University Graduate School of Medical and Dental Sciences

[3]Department of Health Development and Medicine, Osaka University Graduate School of Medicine

[4]Department of Clinical Gene Therapy, Osaka University Graduate School of Medicine

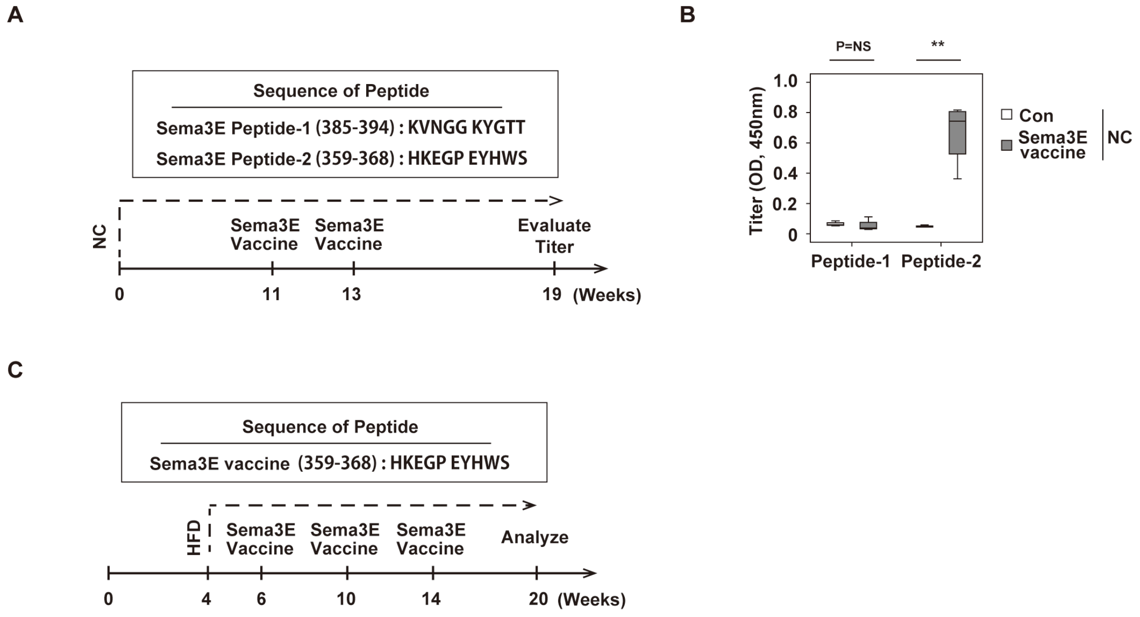

Supplementary Figure 1. Scheme of Sema3E-vaccine therapy, related to Figure 1. (A) Scheme showing the time coarse of Sema3E-vaccine therapy. Wild-type male mice on a C57BL6/NCr background fed a normal chow diet were treated with KLH-conjugated KVNGGKYGTT peptide (Peptide-1) or HKEGPEYHWS peptide (Peptide-2) (20μg) in combination with Freund's adjuvant at 11 and 13 weeks of age. (B) Antibody titers of plasma from mice prepared in Supplementary Figure 1A were evaluated by ELISA at 19 weeks of age (n=3,3,3,4). (C) Scheme showing the time coarse of Sema3E-vaccine therapy. Wild type male mice on a C57BL6/NCr background were fed with a high fat diet since 4 weeks of age. KLH-conjugated HKEGPEYHWS peptide (Sema3E vaccine (20μg)) was administrated for totally 3 times in combination with Freund's adjuvant when mice were 6, 10 or 14 weeks of age. No outlayers or abnormal values were excluded by boxplot analyses. All data were analyzed by the two-tailed Student's *t*-test(B). *$P<0.05$, **$P<0.01$. Values represent the mean ± s.e.m.

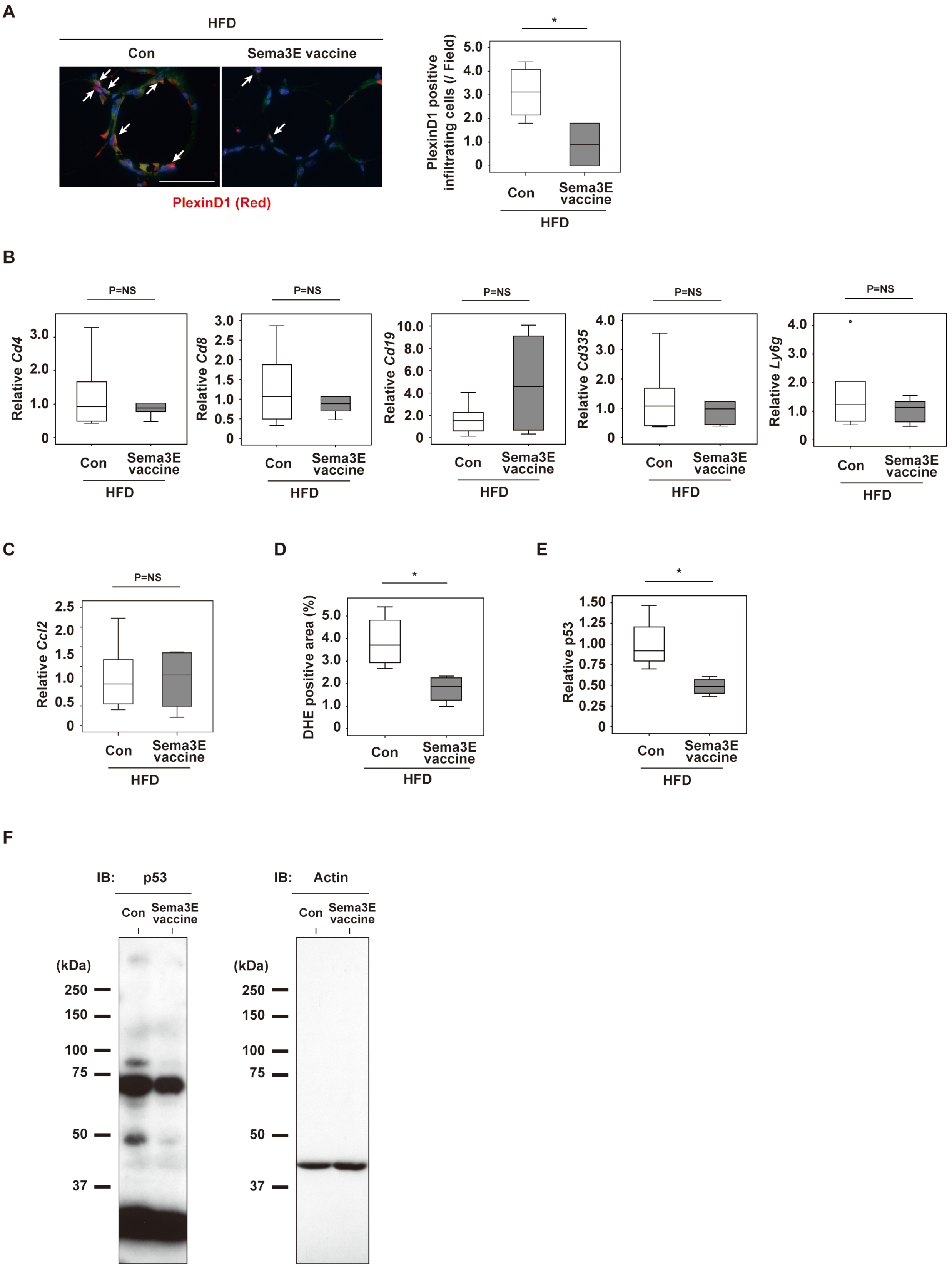
A
HFD
Con
Sema3E vaccine
PlexinD1 (Red)
*
PlexinD1 positive infiltrating cells (/ Field)
5.0
4.0
3.0
2.0
1.0
0
Con
Sema3E vaccine
HFD
B
P=NS
Relative Cd4
Relative Cd8
Relative Cd19
Relative Cd335
Relative Ly6g
Con
Sema3E vaccine
HFD
C
P=NS
Relative Ccl2
D
*
DHE positive area (%)
E
*
Relative p53
F
IB: p53
IB: Actin
Con
Sema3E vaccine
(kDa)
250
150
100
75
50
37

Supplementary Figure 2. The effect of Sema3E-vaccine therapy, related to Figure 1. (A) An immunofluorescent staining for PlexinD1 (red) (scale bar=50 μm) in epididymal WAT (eWAT) harvested from mice in the Sema3E vaccine group (Sema3E vaccine) or the KLH group (Con) maintained on a HFD. Nuclei and plasma membranes were stained with Hoechst (blue) or wheat germ agglutinin (WGA) lectin (green). White arrow shows a PlexinD1-positive infiltrating cell. Right panel indicates the number of PlexinD1-positive infiltrating cells per view (n=4,4). (B) Transcripts for *Cd4* (n=6,5), *Cd8* (n=6,5), *Cd19* (n=5,4), *Cd335* (n=6,5) and *Ly6g* (n=5,5) were evaluated in eWAT from mice in the Sema3E vaccine group (Sema3E vaccine) or KLH group (Con) maintained on a HFD. (C) Quantitative PCR of *Ccl2* in eWAT prepared in Supplementary Figure 2B (n=6,5). (D) Quantification of DHE staining as shown in Fig. 1G (n=4,4). (E) Quantification of p53 relative to actin loading control as shown in Fig. 1H (n=4,4). (F) Full-length blots for p53 expression as presented in Fig. 1H. Outliers were excluded by boxplot analysis. All data were analyzed by the two-tailed Student's *t*-test (A-E). *$P<0.05$, **$P<0.01$. Values represent the mean ± s.e.m. NS = not significant.
